# Fluorescent sensor-modified polyvinyl alcohol films for the detection of amine vapor based on PET (photo-induced electron transfer)

**DOI:** 10.1039/d5ra05520b

**Published:** 2025-09-30

**Authors:** Kazuki Tao, Keiichi Imato, Yousuke Ooyama

**Affiliations:** a Applied Chemistry Program, Graduate School of Advanced Science and Engineering, Hiroshima University 1-4-1 Kagamiyama Higashi-Hiroshima 739-8527 Japan yooyama@hiroshima-u.ac.jp

## Abstract

Biogenic volatile amine vapors are released during food spoilage. In this work, we investigated the fluorescent sensing properties of an intramolecular photo-induced electron transfer (intraPET)-type fluorescent sensor, TF-2, composed of anthracene-AminoMeCNPhenylB(OH)_2_, for amine. TF-2 solutions in the absence of amines show strong fluorescence emission due to the formation of intraPET inactive (fluorescent) species with the intramolecular OH⋯N hydrogen bonding between the nitrogen atom of the amino moiety and the hydroxyl group of the B(OH)_2_ moiety. Moreover the TF-2 solutions caused a decrease in the fluorescence intensity upon the addition of amines, which is attributed to the intermolecular PET (interPET) from the amine to TF-2. Moreover, we prepared a TF-2-modified polyvinyl alcohol (PVA) film for detecting volatile amines. It was found that the TF-2-modified PVA film upon exposure to amine vapors shows a drastic decrease in the fluorescence intensity due to the interPET from the amine to the TF-2. Finally, we monitored changes in the fluorescence emission of the TF-2-modified PVA film upon exposure to volatile amine vapors released from the decomposing matter during the spoilage of raw shrimp. Here, we propose that the PET-type fluorescent sensor based on a fluorescence quenching (turn-off) system is one of the most promising and useful functional dye materials for detecting organic amines.

## Abstract 2

The photo-induced electron transfer (PET)-type fluorescent sensor TF-2-modified polyvinyl alcohol (PVA) film can be used as a fluorescent material for the detection of volatile amines released from decomposing matter during food spoilage.

## Graphical abstract

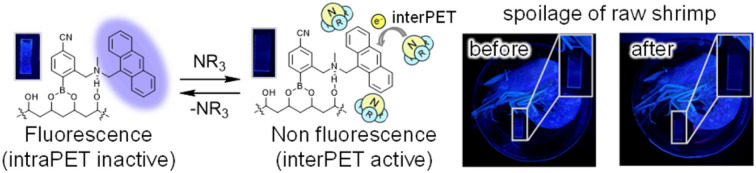

## Introduction

Organic amines are widely used in dyeing, polymer, pharmaceutical, cosmetics, and agrochemical industries.^1,2^ Biogenic volatile amine vapors are released by the microbial enzymatic decarboxylation of amino acids during meat, fish and food spoilage.^3–5^ Most volatile organic amines are toxic, irritant, and corrosive to the human eyes, skin and respiratory system.^6,7^ Therefore, the detection of volatile organic amines is indispensable for protecting human health as well as for monitoring industrial and environmental pollution. Indeed, several analytical methods have been developed for the detection of organic amines, including gas chromatography-mass spectrometry (GC-MS),^8^ electrochemistry,^9^ and high-performance liquid chromatography (HPLC).^10^ Although these methods are sufficiently accurate, they generally require time-consuming sample-preparation procedures, advanced experimental skills for operation and analysis, and expensive instrumentation. On the contrary, fluorescence detection methods based on fluorescent dyes, which are simple to operate and analyse, not only exhibit sufficient accuracy, high sensitivity and fast response but also allow visualization and real-time monitoring of organic amines. Actually, various types of fluorescent sensors for organic amines have been developed: chemodosimeters, which are based on the chemical reaction of fluorophores with amines, including decomposition,^11^ Schiff base formation,^12–17^ aminolysis reaction,^18–23^ nucleophilic addition,^24–27^ substitution reaction,^28^ amine exchange,^29^ and cyclization;^30–32^ indicators based on the deprotonation of fluorophores with amines,^33,34^ formation of hydrogen bonds,^35^ exciplex formation,^36^ and intramolecular charge transfer (ICT);^37^ and intermolecular photo-induced electron transfer (interPET)-type sensors based on electron transfer from amine molecules to the photoexcited fluorophore,^38–51^ leading to changes in wavelength, intensity, and lifetime of fluorescence emission in the presence of organic amines. Among them, in particular, PET-type fluorescent sensors that can recognize organic amines without a chemical reaction, that is, without a change in the chemical structure of a fluorophore, have attracted significant attention as practical sensors for detecting amine vapor due to their higher sensitivity and fast response.

**Fig. 1:**
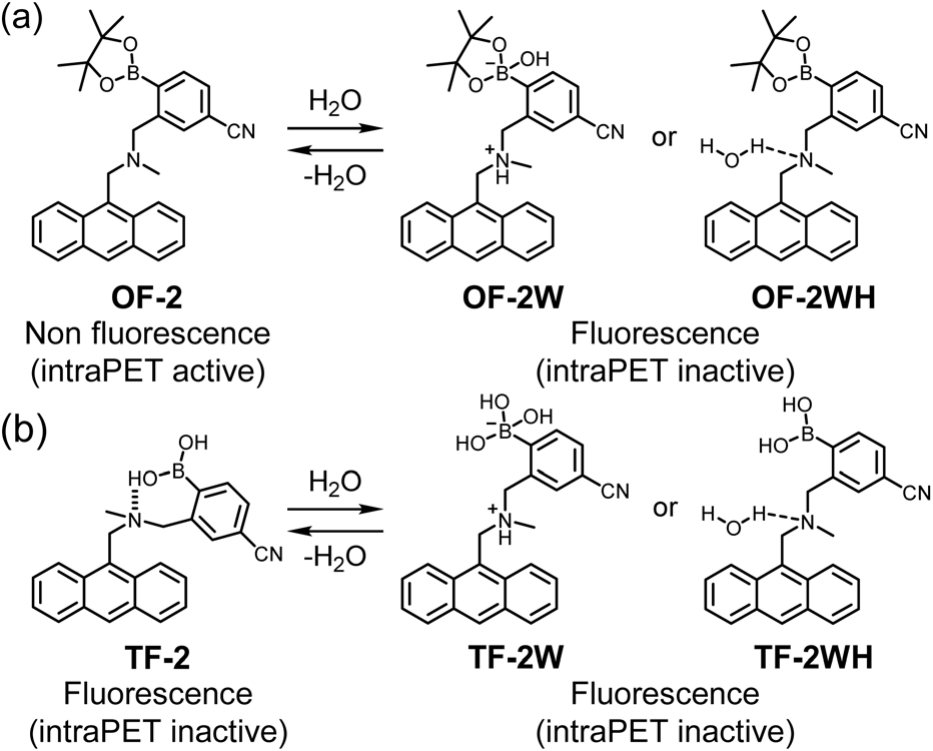
Mechanisms of intraPET-type fluorescent sensors (a) OF-2 and (b) TF-2 for the detection of water in organic solvents (our previous work).^52,57^

In our previous work, we have designed and developed anthracene-(aminomethyl)-4-cyanophenylboronic pinacol esters (AminoMeCNPhenylBPin) OF-2 as intramolecular PET (intraPET)-type fluorescent sensors for determining trace amounts of water in solvents (Fig. 1a).^52^ In OF-2, the intraPET occurs from the nitrogen atom of the amino moiety to the photoexcited anthracene skeleton in the absence of water, resulting in fluorescence quenching (PET active state). The addition of water to solvents containing OF-2 causes a drastic and liner enhancement of fluorescence emission as a function of water content, which is due to the inhibition of intraPET; the nitrogen atom of the amino moiety is protonated or strongly interacts with water molecules, resulting in the formation of PET inactive (fluorescent) species such as the zwitterionic structure OF-2W or the hydrogen-bonded structure OF-2WH. Moreover, we have prepared an OF-2-doped polymer film,^53,54^ a copolymer film composed of an OF-2 derivative having a methyl methacrylate group (MMA),^55^ and an OF-2-immobilized glass substrate,^56^ which produced sufficient reversible fluorescence off–on switching between the intraPET active state, formed upon drying, and the intraPET inactive state, when exposed to moisture or water droplets. Consequently, in our previous work, we proposed that the intraPET-type fluorescence monomer OF-2 and films containing it are one of the most promising and convenient functional dye materials for the visualization and detection of water. More recently, we found that a solution of anthracene-AminoMePhenylB(OH)_2_TF-2, which has a B(OH)_2_ moiety as a substitute for BPin, exhibits intense fluorescence emission, even in the absence of water, due to the formation of intraPET inactive (fluorescent) species with intramolecular OH⋯N hydrogen bonding between the nitrogen atom of the amino moiety and the hydroxyl group of B(OH)_2_ moiety (Fig. 1b).^57^ Thus, the TF-2 solution showed little change in the fluorescence intensity upon the addition of water, even though the PET inactive (fluorescent) species TF-2W or TF-2WH were formed by interaction with water molecules. Meanwhile, we serendipitously discovered that the TF-2 solutions caused a drastic decrease in fluorescence intensity upon the addition of triethylamine. In contrast to the fluorescence enhancement (turn-on) system based on intraPET-type fluorescent sensors developed so far, these interesting results inspired us to conceive the construction of a fluorescence quenching (turn-off) system based on intraPET-type fluorescent sensors for detecting organic amines.

Thus, in this work, we investigated the fluorescent sensing abilities of TF-2 for a series of organic amines. Moreover, we prepared a TF-2-modified polyvinyl alcohol (PVA) film that can act as a functional dye material for detecting volatile organic amines based on the fluorescence quenching (turn-off) system. Indeed, it was found that the TF-2-modified PVA film shows a drastic decrease in fluorescence intensity upon exposure to amine vapors, due to interPET from the amine to TF-2. Finally, we tested the change in fluorescence emission of TF-2-modified PVA film upon exposure to volatile amine vapors released from decomposing matter during the spoilage of raw shrimp as an example. Here, we propose that a PET-type fluorescent sensor based on a fluorescence quenching (turn-off) system is one of the most promising and useful functional dye materials for detecting volatile organic amines.

## Results and discussion

### Optical sensing ability of TF-2 for organic amines in solvents

**Fig. 2:**
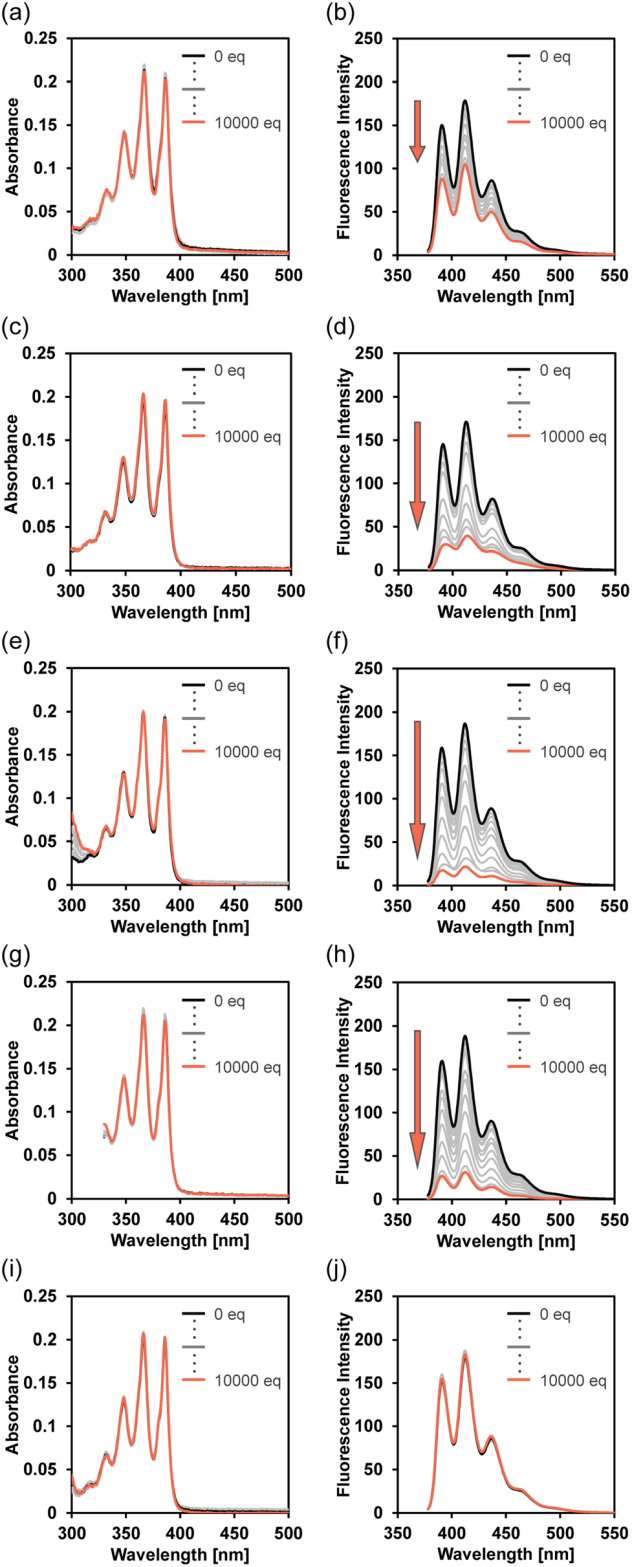
Photoabsorption spectra of TF-2 (2.0 × 10^−5^ M) in acetonitrile containing (a) BA (0–0.2 M), (c) DEA (0–0.2 M), (e) TEA (0–0.2 M), (g) AN (0–0.2 M), and (i) PY (0–0.2 M). Fluorescence spectra (*λ*^ex^ = 367 nm) of TF-2 (2.0 × 10^−5^ M) in acetonitrile containing (b) BA (0–0.2 M), (d) DEA (0–0.2 M), (f) TEA (0–0.2 M), (h) AN (0–0.2 M), and (j) PY (0–0.2 M).

The optical sensing ability of TF-2 for categories of organic amines, including butylamine (BA), diethylamine (DEA) and triethylamine (TEA), as aliphatic primary, secondary, and tertiary amines, respectively, 1,5-pentadiamine (PDIA, *i.e.* cadaverine known as a putrefactive amine), as a diamine, aniline (AN), as an aromatic amine, and pyridine (PY), as a heterocyclic aromatic amine, was investigated by photoabsorption and fluorescence spectral measurements of TF-2 (2.0 × 10^−5^ M) in acetonitrile containing various concentrations (0–0.2 M; 0−10 000 equiv.) of amine (Fig. 2). For all six amines, the TF-2 solution exhibited a vibronically structured photoabsorption band in the range of 300 nm to 400 nm derived from the anthracene skeleton, and no significant changes in the absorbance or shape were observed upon addition of amines to the solutions (Fig. 2a, c, e, g and i, see Fig. S1a for PDIA). For the corresponding fluorescence spectra, the TF-2 solution without added amines exhibited an intense and vibronically-structured fluorescence band with a fluorescence maximum wavelength (*λ*^fl^_max_) at 412 nm in the range of 400 nm to 500 nm, which is due to the monomer emission arising from the anthracene fluorophore in the intraPET inactive state (Fig. 2b, d, f, h and j, see Fig. S1b for PDIA). When amines were added to the solution of TF-2, a decrease in fluorescence intensity was observed. It is worth noting here that there is a big difference in the degree of fluorescence quenching between the amines. Indeed, the fluorescence intensity of TF-2 solution showed a significant decrease upon the addition of DEA, TEA, and AN, and a moderate decrease upon the addition of BA and PDIA. However, there was no change in the fluorescence intensity of the TF-2 solution upon the addition of PY.

**Fig. 3:**
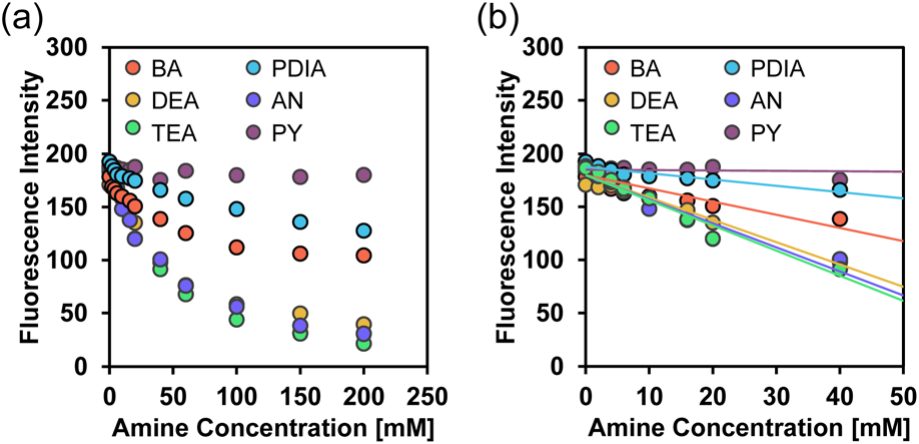
Fluorescence peak intensity around 412 nm of TF-2 (*λ*^ex^ = 367 nm) as a function of amine content below (a) 2.0 × 10^−1^ M and (b) 4.0 × 10^−2^ M in acetonitrile.

Thus, to clarify the sensitivity and accuracy of TF-2 in detecting organic amines in solvents, the changes in the fluorescence peak intensity at around 412 nm were plotted against the amine concentration in solvents (Fig. 3), and the slope (*m*_s_) was used to estimate the detection limit (DL): DL = 3.3*σ*/*m*_s_, where *σ* is the standard deviation of the blank sample and *m*_s_ is the slope of the calibration curve obtained from the plot of the fluorescence peak intensity at around 412 nm *versus* the amine fraction in the amine concentration range below 2000 equiv. (4.0 × 10^−2^ M) (Fig. 3b). It was found that the slopes for DEA, TEA, and AN indicated a dramatic and linear decrease in fluorescence peak intensity, while the plots for BA and PDIA exhibited moderate slopes. On the other hand, the plot for PY had a negligibly small slope, indicating non-responsivity of TF-2 to PY. The calibration curves for BA, DEA, TEA, PDIA, and AN exhibited good linearity, with correlation coefficients (*R*^2^) of 0.92, 0.99, 0.96, 0.88, and 0.92, respectively. In contrast, the calibration curve for PY showed a lower *R*^2^ value of 0.39, which may be attributed to the small change in fluorescence peak intensity. The *m*_*s*_ values of the calibration curves for DEA, TEA, and AN are −2.11 × 10^3^, −2.37 × 10^3^, and −2.27 × 10^3^, respectively, which are larger than those for BA and PDIA (−1.24 × 10^3^ and −5.94 × 10^2^, respectively), and are much larger than that for PY (−3.62× 10^1^) (Table 1). Consequently, the DLs of TF-2 were estimated to be 2.66 mM for BA, 1.56 mM for DEA, 1.39 mM for TEA, 5.56 mM for PDIA, 1.45 mM for AN, and 91.2 mM for PY, indicating that TF-2 possesses a superior sensitivity to DEA, TEA, and AN. However, the DLs of TF-2 for amines are inferior to those (μM) of the reported interPET-type fluorescent sensors.^43,46^ This result may be due to the low fluorescence quantum yield (*Φ*_fl_ = 0.19) of TF-2.^57^

**Table 1:** DLs of TF-2 for amines in acetonitrile

Sensor	Amine	*m*_s_ a	DL/mM^b^
TF-2	BA	−1.24 × 10^3^	2.66
	DEA	−2.11 × 10^3^	1.56
	TEA	−2.37 × 10^3^	1.39
	PDIA	−5.94 × 10^2^	5.56
	AN	−2.27 × 10^3^	1.45
	PY	−3.62 × 10^1^	91.2

a Slope (*m*_s_) of the calibration curve obtained from plots of the fluorescence peak intensity (*ca.* 412 nm) of TF-2 (2.0 × 10^−5^ M) *versus* the amine fraction in the amine content region below 4.0 × 10^−2^ M in acetonitrile.

b Detection limit (DL) of TF-2 for amines.

**Fig. 4:**
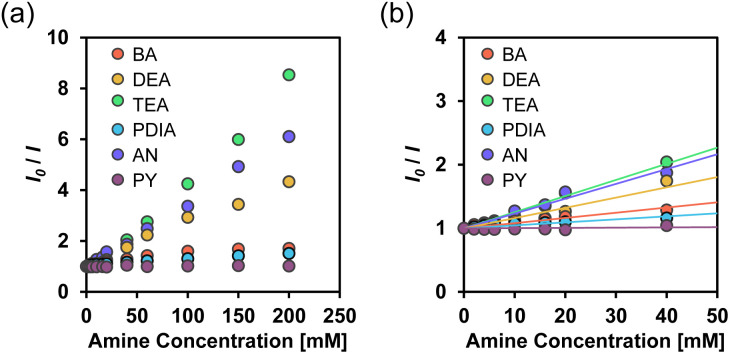
Stern–Volmer plot for acetonitrile solutions of TF-2 (2.0 × 10^−5^ M) with amine contents below (a) 2.0 × 10^−1^ M and (b) 4.0 × 10^−2^ M.

**Fig. 5:**
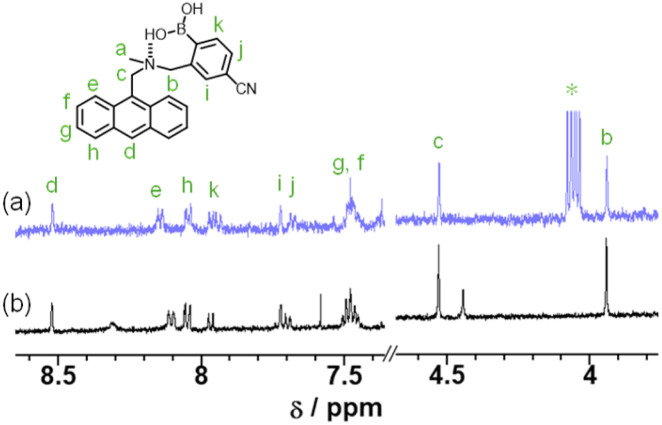
^1^H NMR spectra of TF-2 (2.0 × 10^−5^ M) in acetonitrile-*d*_3_ (a) with and (b) without the addition of TEA-*d*_15_ (2.0 × 10^−1^ M). The proton H_a_ was omitted from the spectra because it overlaps with the proton signal of water. The signal * is the methylene proton of TEA.

Fluorescence quenching induced by quenchers can be mainly classified into two mechanisms, static and dynamic quenching, depending on the interaction between the fluorophore and the quencher. Static quenching occurs as a result of the association between the fluorophore and the quencher in the ground state to form the non-fluorescent complex. Dynamic quenching occurs as a result of nonradiative deactivation by collision between the fluorophore in the excited state and the quencher. Thus, in order to estimate the fluorescence quenching (turn-off) mechanism of the PET-type fluorescent sensor by amines, the fluorescence quenching dynamics were investigated by the following Stern–Volmer (SV) relationship [eqn (1)]1
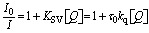
where *I*_0_ and *I* are the fluorescence intensities in the absence and presence of the quenchers (Q = amines), respectively, *K*_SV_ is the SV quenching constant in M^−1^ containing static and dynamic quenching components or association constant in M^−1^ for static quenching, *τ*_0_ is the fluorescence lifetime of the fluorophores in the absence of quenchers, *k*_q_ is the bimolecular quenching rate constant in M^−1^ s^−1^, and [*Q*] is the concentration of the quenchers. The SV plots of the change (*I*_0_/*I*) in fluorescence intensity of TF-2 (2.0 × 10^−5^ M) upon addition of amines in the acetonitrile are shown in Fig. 4. The SV plots for DEA, TEA, and AN have steep positive slopes with good linearity, with *R*^2^ values of 0.96–0.99 over amine concentration regions below 2.0 × 10^−1^ M and 4.0 × 10^−2^ M. On the other hand, the SV plots for BA and PDIA have a more gradual positive slope, and the plot for PY has an almost plateau response in the amine concentration region below 2.0 × 10^−1^ M. Thus, the *K*_SV_ values for the amines were estimated to be 8.1 M^−1^ for BA, 16 M^−1^ for DEA, 25 M^−1^ for TEA, 4.7 M^−1^ for PDIA, 23 M^−1^ for AN, and 0.3 M^−1^ for PY from the slopes of the SV plots (Fig. 4b and Table 2). In addition, the *k*_q_ values were determined to be 3.6 × 10^9^ M^−1^ s^−1^ for BA, 7.1 × 10^9^ M^−1^ s^−1^ for DEA, 1.1 × 10^10^ M^−1^ s^−1^ for TEA, 2.1 × 10^9^ M^−1^ s^−1^ for PDIA, 1.0 × 10^10^ M^−1^ s^−1^ for AN, and 1.3 × 10^8^ M^−1^ s^−1^ for PY from the *K*_SV_ values and the fluorescence lifetime (*τ*_0_ = 2.27 ns) of TF-2 in the absence of amines obtained by time-resolved fluorescence spectroscopy. This result shows that there is a little difference in the *K*_SV_ and *k*_q_ values between DEA, TEA, and AN. On the other hand, the *K*_SV_ and *k*_q_ values for BA and PDIA are lower than those for DEA, TEA, and AN, and those for PY are significantly lower than those for the other five amines. Here, one can see that the *K*_SV_ and *k*_q_ values for aliphatic and aromatic amines are higher than those for the heterocyclic aromatic amine. Thus, we examined the difference in fluorescence quenching efficiency among amines based on acid dissociation constant (p*K*_a_) values^58,59^ for amines. The p*K*_a_ values for BA, DEA, TEA, PDIA, AN, and PY are 10.7, 10.9, 10.8, 10.3, 4.60, and 5.21, respectively, indicating that there is no correlation between the *K*_SV_ and *k*_q_ values and the p*K*_a_ values for amines. In order to estimate the fluorescence quenching (turn-off) mechanism of TF-2 by the amines with a focus on ground–state complex formation, we recorded ^1^H NMR spectra of TF-2 in acetonitrile-*d*_3_ with and without the addition of triethylamine-*d*_15_ (TEA-*d*_15_). One can see that there is no obvious change in the chemical shift or splitting of any of the signals in the ^1^H NMR spectrum of TF-2 upon the addition of TEA-*d*_15_ (Fig. 5), indicating that TF-2 does not form associates or undergo reactions with the added amines. This result indicates that the static fluorescence quenching mechanism based on the non-fluorescent complex formation between TF-2 and amine molecules in the ground state can be ruled out.

**Table 2:** *K*_sv_ and *k*_q_ values from Stern–Volmer plots and fluorescence lifetimes of TF-2 in acetonitrile with and without the addition of amines

Sensor	Amine	*K*_SV_ a/M^−1^	*k*_q_ a/M^−1^ s^−1^	*τ*/ns
TF-2	—	—	—	2.27^b^
	BA	8.1	3.6 × 10^9^	1.92^c^
	DEA	16	7.1 × 10^9^	0.82^c^
	TEA	25	1.1 × 10^10^	0.55^c^
	PDIA	4.7	2.1 × 10^9^	1.81^c^
	AN	23	1.0 × 10^10^	0.35^c^
	PY	0.3	1.3 × 10^8^	2.16^c^

a *K*_sv_ and *k*_q_ values obtained from Stern–Volmer plots for acetonitrile solutions of TF-2 (2.0 × 10^−5^ M) containing amine concentrations below 4.0 × 10^−2^ M.

b *τ* value of the TF-2 solution in the absence of amine.

c *τ* value of the TF-2 solution with an amine concentration of 2.0 × 10^−1^ M.

Meanwhile, fluorescence lifetime measurements are a useful way to distinguish between dynamic and static quenching, because the fluorescence lifetime changes upon addition of a quencher in dynamic quenching, but remains unchanged upon addition of a quencher in static quenching. Thus, we performed time-resolved fluorescence spectroscopy to determine the fluorescence lifetimes (*τ*) of TF-2 (2.0 × 10^−5^ M) in the presence of amines in the concentration range of 2.0 × 10^−5^ to 2.0 × 10^−1^ M in acetonitrile (Fig. S2). It was found that the *τ* value of TF-2 is 2.27 ns in the absence of amine, but decreases dramatically upon the addition of DEA, TEA, and AN (*τ* = 0.35–0.82 ns) and decreases slightly with the addition of BA and PDIA (*τ* = 1.92 ns and 1.81 ns, respectively), indicating dynamic quenching by the interPET from amines to TF-2 (Table 2). However, the *τ* value of TF-2 remains unchanged with the addition of PY (*τ* = 2.16 ns). In contrast, for the intraPET-type fluorescent sensor OF-2 for water based on the fluorescence enhancement (turn-on) system, the *τ* value of the OF-2 did not change before (2.39 ns) and after (2.55 ns) the addition of water to the acetonitrile solution, which is due to the formation of the intraPET inactive (fluorescent) species (OF-2W or OF-2WH) between OF-2 and water molecules in the ground state (Fig. 1a). The result also confirms that the mechanism of TF-2 detection of amines is attributable to dynamic fluorescence quenching based on interPET from the amine to the photoexcited TF-2.

**Fig. 6:**
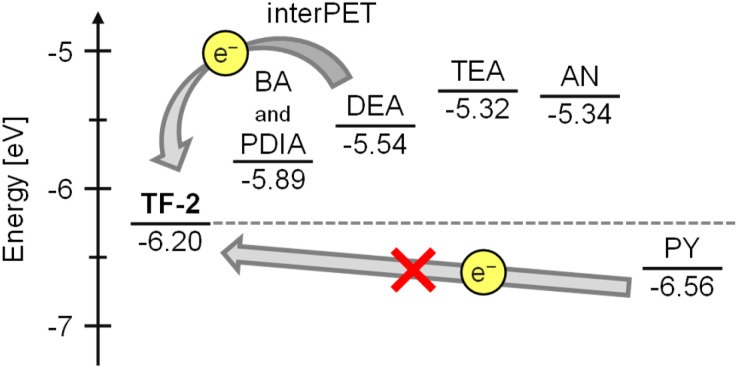
Energy level diagram for the HOMO of TF-2 and amines estimated from the oxidation peak potential (*E*^ox^_pa_).^59–63^ For the HOMO energy levels of BA and PDIA, the *E*^ox^_pa_ of penthylamine was used as a substitute for those of BA and PDIA.

**Fig. 7:**
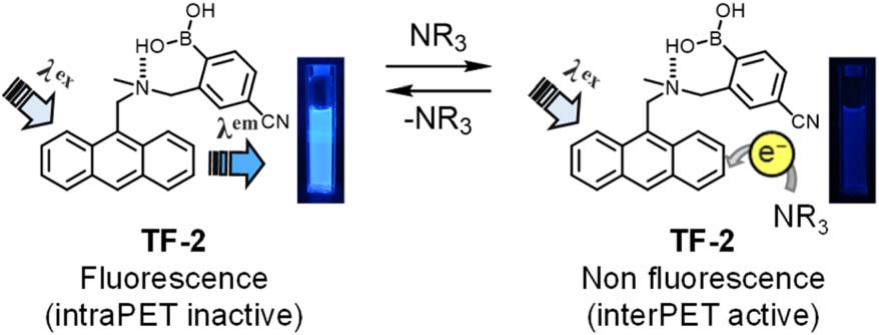
Mechanism of TF-2 for the detection of amines. Photographs show acetonitrile solutions of TF-2 (under 365 nm irradiation) before and after the addition of TEA.

Furthermore, in order to examine the thermodynamic probability of interPET from amine molecules to the photoexcited fluorophores, which is responsible for the dynamic quenching mechanism, the highest occupied molecular orbital (HOMO) energy level of TF-2 was estimated from the oxidation peak potential (*E*^ox^_pa_). This was obtained by cyclic voltammetry (CV) with an acetonitrile solution (1.0 × 10^−3^ M) of TF-2 containing 0.1 M tetrabutylammonium perchlorate (Bu_4_NClO_4_). The cyclic voltammogram showed an irreversible oxidation wave at around 1.0–1.4 V with an oxidation peak potential (*E*^ox^_pa_) at 1.20 V *versus* Ag/Ag^+^, corresponding to oxidation of the anthracene skeleton (Fig. S3a). The *E*^ox^_pa_ of TF-2 was converted to 1.76 V *versus* the saturated calomel electrode (SCE), because the *E*^ox^_pa_ values of amines *versus* SCE were obtained from the ref. 60–64. Thus, the HOMO energy levels (–[*E*^ox^_pa_ + 4.44] eV) *versus* vacuum level were estimated to be –6.20 eV for the anthracene skeleton of TF-2, –5.89 eV for BA, –5.54 eV for DEA, –5.32 eV for TEA, –5.89 eV for PDIA, –5.34 eV for AN, and –6.56 eV for PY from the *E*^ox^_pa_. For the HOMO energy levels of BA and PDIA the *E*^ox^_pa_ of penthylamine was used as a substitute for that of BA and PDIA (Fig. 6). Indeed, the HOMO energy levels of DEA, TEA, and AN are much higher than that of TF-2, thus ensuring that the interPET from the amine molecules to the photoexcited TF-2 is thermodynamically feasible. Meanwhile, the HOMO energy levels of BA and PDIA are slightly higher than that of TF-2 by *ca.* 0.3 eV, indicating that the efficiency of interPET for BA and PDIA is inferior to that for DEA, TEA, and AN. Obviously, the HOMO energy level of PY is lower than that of TF-2; thus, the interPET for PY is thermodynamically impossible. In order to confirm the dynamic fluorescence quenching based on the interPET, we performed photoabsorption and fluorescence spectral measurements of TF-2 (2.0 × 10^−5^ M) in acetonitrile containing various concentrations (0–0.2 M; 0−10 000 equiv.) of anisole or 1,2,4-trimethoxybenzene (Fig. S4), where anisole (−6.20 eV) and 1,2,4-trimethoxybenzene (−5.56 eV) were used as guests with HOMO energy levels similar to and much higher than that of TF-2 (−6.20 eV), respectively. The photoabsorption spectra did not undergo appreciable changes in shape and absorbance upon the addition of anisole and 1,2,4-trimethoxybenzene. On the other hand, it was found that the fluorescence intensity of the TF-2 solution decreased upon the addition of 1,2,4-trimethoxybenzene. However, there was no change in the fluorescence intensity of the TF-2 solution upon the addition of anisole. Consequently, these results strongly support the conclusion that the mechanism of the PET-type fluorescent sensor TF-2 for the detection of amines is attributable to dynamic fluorescence quenching based on the interPET from the amine to photoexcited TF-2 (Fig. 7).

### Optical sensing ability of the TF-2-modified PVA film for volatile organic amines

**Fig. 8:**
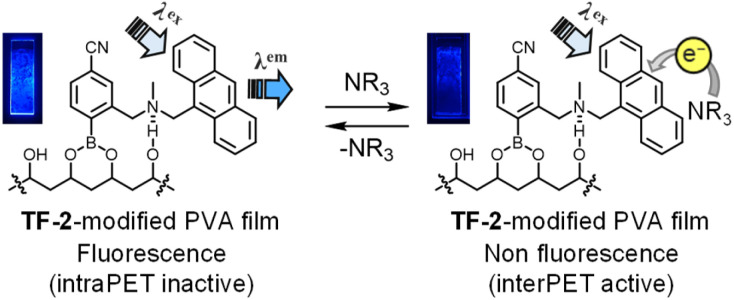
Mechanism of the TF-2-modified PVA film for the detection of amine vapor. Photographs show the TF-2-modified PVA film (under 365 nm irradiation) before and after exposure to TEA vapors.

Kubo *et al.* reported that 1,4-phenylenediboronic acid (DBA) serves as an efficient crosslinking agent for the attachment of compounds to the surface of solid polyvinyl alcohol (PVA) through boronate esterification.^65^ Thus, we prepared a PVA film with TF-2 and DBA by chemical modification. It was found that the as-prepared TF-2-modified PVA film exhibits strong fluorescence emission due to the formation of intraPET-inactive species because of the intramolecular OH⋯N hydrogen bonding between the hydroxyl group of PVA and the amino moiety of TF-2. Actually, the TF-2-modified PVA film exhibited visual blue emission under 365 nm irradiation (Fig. 8). When the TF-2-modified PVA film was exposed to TEA vapor, the blue emission weakened due to the interPET from TEA molecules to the photoexcited TF-2 molecule. In fact, the ^1^H NMR spectrum of TF-2 in ethanol-*d*_6_ suggested that the PET-inactive (fluorescent) species was formed by intermolecular OH⋯N hydrogen bonding between the hydroxyl group of ethanol and the amino moiety of TF-2, and it remains unchanged with the addition of TEA-*d*_15_ (Fig. S5). Moreover, the fluorescence spectral and lifetime measurements demonstrated that the fluorescence intensity and the *τ* value (2.11 ns) of TF-2 in ethanol decrease with the addition of TEA (*τ* = 0.97 ns), but remains almost unchanged with the addition of PY (*τ* = 2.72 ns) (Fig. S6 and Table S1), as in the case of the acetonitrile solution. The result indicates that the fluorescence quenching (turn-off) system of the TF-2-modified PVA film for the detection of amine vapor is based on a change from the intraPET inactive state between PVA and TF-2 to the interPET active state between amine and TF-2 (Fig. 8).

**Fig. 9:**
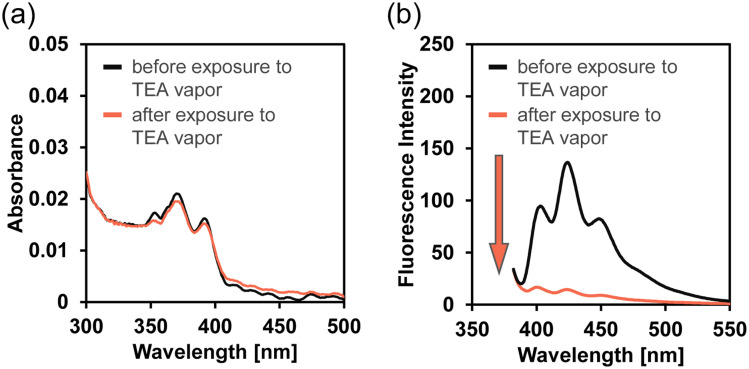
(a) Photoabsorption and (b) fluorescence spectra (*λ*^ex^ = 367 nm) of the TF-2-modified PVA film before and after exposure to TEA vapors.

**Fig. 10:**
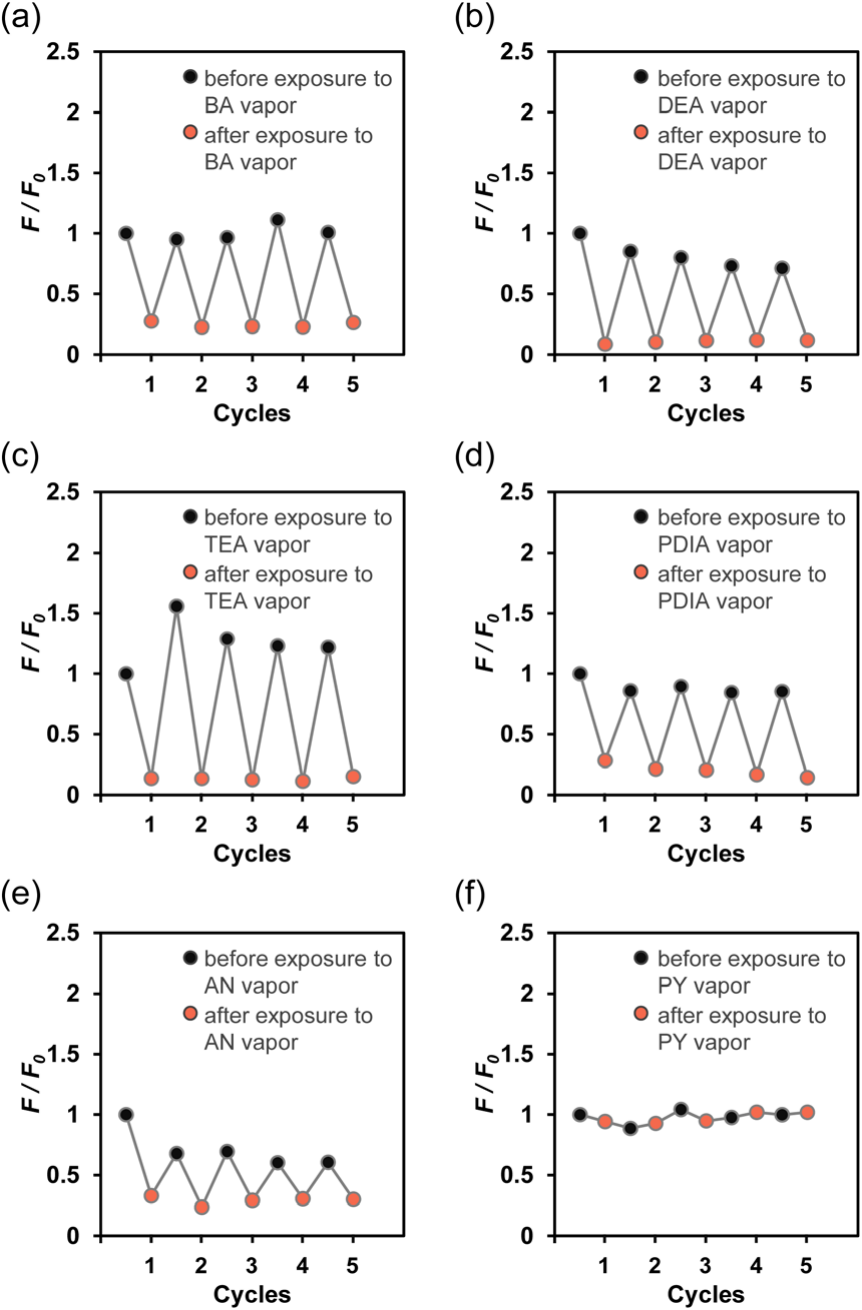
Reversible switching of the relative fluorescence intensity (*F*/*F*_0_) around 422 nm for the TF-2-modified PVA film in air and (a) BA, (b) DEA, (c) TEA, (d) PDIA, (e) AN, and (f) PY-vapor cycles. *F*_0_ is the fluorescence intensity of the as-prepared TF-2-modified PVA film.

Therefore, in order to evaluate the fluorescence quenching of TF-2-modified PVA film upon exposure to amine vapors, we recorded photoabsorption and fluorescence spectra of TF-2-modified PVA film before and after exposure to amine vapors. The as-prepared TF-2-modified PVA film showed a vibronically-structured photoabsorption band in the range of 300 nm to 400 nm and a monomer fluorescence band with *λ*^fl^_max_ at 422 nm in the range of 400 nm to 500 nm originating from the anthracene fluorophore in the PET inactive state. For TF-2-modified PVA film after exposure to BA, DEA, TEA, PDIA, AN, or PY vapor, the photoabsorption spectra did not undergo appreciable changes in shape or absorbance (Fig. 9a for TEA, see Fig. S7a, c, g, i, and k for the other amines). The corresponding fluorescence spectra after exposure to BA, DEA, TEA, PDIA, and AN vapors, except PY vapor, showed a decrease in the fluorescence intensity (Fig. 9b for TEA, see Fig. S7b, d, h, j, and l for the other amines). It is worth noting here that when the TF-2-modified PVA films were placed in air (drying process) after exposure to amine vapors, the fluorescence spectrum recovered its original spectral shape and intensity to that seen before exposure to amine vapors. Thus, in order to clarify the difference in the magnitude of the change in the fluorescence intensity as well as its reversibility in air–amine vapor cycle between amine vapors, the changes in the fluorescence intensity at *λ*^fl^_max_ (*ca.* 422 nm) were plotted against the air–amine vapor cycle processes (Fig. 10). It was found that for BA, DEA, TEA, PDIA, and AN vapors, except for PY vapors, the TF-2-modified PVA films showed a good reversible switching of the fluorescence intensity through five air–amine vapor cycles. Furthermore, it is worth mentioning here that the average ratio of the fluorescence intensity of the air process to the amine vapor process (*F*_air_/*F*_amine_) in the five air–amine vapor cycles is 7.8 for DEA and 9.5 for TEA. These values are significantly higher than those for BA and PDIA vapors (4.1 and 4.6, respectively), indicating that the TF-2-modified PVA film exhibits a high sensitivity to DEA and TEA vapors, as with the case of the TF-2 solution, although the *F*_air_/*F*_amine_ value for AN (2.5) is the smallest, which may be due to the interaction of AN with DBA as crosslinking agent for PVA.

**Fig. 11:**
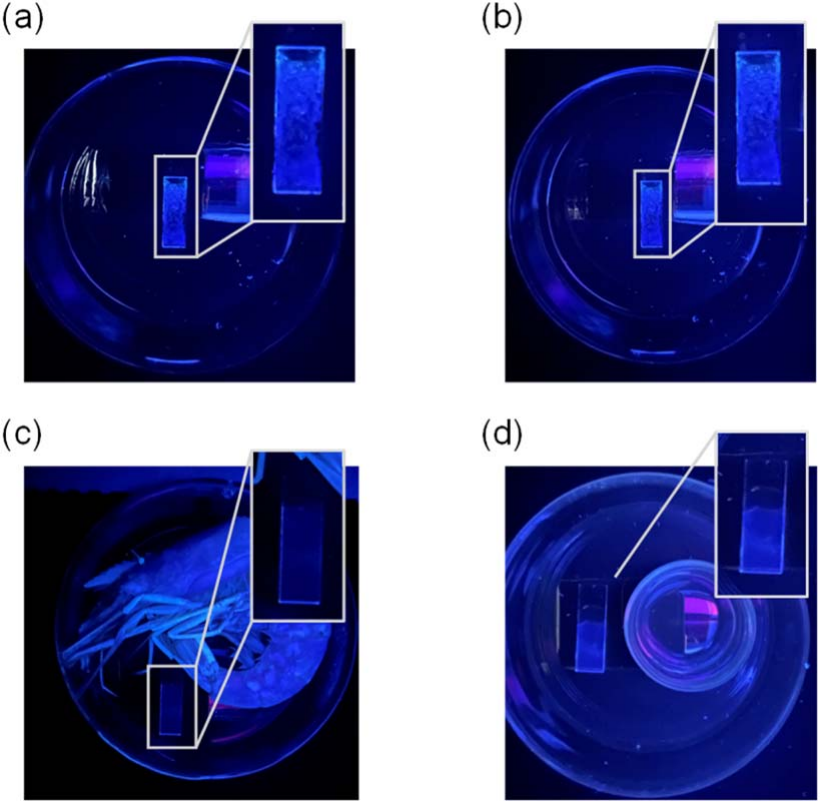
Photographs (under 365 nm irradiation) of the (a) as-prepared TF-2-modified PVA film, (b) TF-2-modified PVA film after exposure to air, (c) TF-2-modified PVA film during spoilage of a raw shrimp, and (d) TF-2-modified PVA film after exposure to moisture at 25 °C for 24 h.

Biogenic volatile amine vapors, including TEA and cadaverine (PDIA) are released by microbial enzymatic decarboxylation of amino acids during meat, fish, and food spoilage. Therefore, we monitored the change in fluorescence emission of TF-2-modified PVA film under 365 nm irradiation during the spoilage of raw shrimp as an example. When raw shrimp and TF-2-modified PVA film were sealed in a Petri dish and stored at 25 °C, the blue emission of TF-2-modified PVA film was quenched after 24 h (Fig. 11a and c). On the other hand, the blue emission of TF-2-modified PVA film remained unchanged after being exposed to air or moisture for 24 h (Fig. 11b and d). This fact demonstrates that TF-2-modified PVA film can be used as a fluorescent sensor for detecting volatile amine vapors released from decomposing matter during food spoilage. Consequently, we have achieved the preparation of a PET-type fluorescent sensor-modified PVA film that can act as a functional dye material for detecting volatile organic amines based on a fluorescence quenching (turn-off) system.

## Conclusions

We investigated the fluorescent sensing properties of an intramolecular photo-induced electron transfer (intraPET)-type fluorescent sensor TF-2 composed of anthracene-AminoMeCNPhenylB(OH)_2_ for categories of organic amines, including aliphatic amines, aromatic amines, and a heterocyclic aromatic amine. It was revealed that the mechanism of the PET-type fluorescent sensor TF-2 for the detection of amines is attributable to the dynamic fluorescence quenching based on intermolecular PET (interPET) from the amine to photoexcited TF-2; the interPET from amine molecules to the photoexcited TF-2 is thermodynamically feasible when the HOMO energy levels of amines are much higher than that of TF-2. Moreover, we demonstrated that TF-2-modified PVA film can be used as a fluorescent sensor for efficient detection of volatile amine vapors released from decomposing matter during food spoilage. Consequently, this work offers a PET-type fluorescent sensor based on a fluorescence quenching (turn-off) system as one of the most promising and convenient functional dye materials for detecting volatile organic amines.

## Author contributions

Y. O. conceived the project and directed the experimental work. K. I. was responsible for the preparation and analysis of the polymer film. K. T. performed most of the experiments and performed the DFT calculations. The manuscript was written with contributions of all authors.

## Conflicts of interest

The authors declare that there are no conflicts of interest.

## Supplementary Material

RA-015-D5RA05520B-s001

## Data Availability

The data supporting this article have been included as part of the supplementary information (SI). Supplementary information is available. See DOI: https://doi.org/10.1039/d5ra05520b.
