# Supplementary material for: Fluorescent sensor-modified polyvinyl alcohol films for the detection of amine vapor based on PET (photo-induced electron transfer)
Source: RSC Adv. 2025 Sep 30;15(43):36165–74. doi: 10.1039/d5ra05520b (PMC12481236; doi:10.1039/d5ra05520b)
Supplement: RA-015-D5RA05520B-s001 [file RA-015-D5RA05520B-s001.pdf]

Supplementary Information (SI) for RSC Advances.
This journal is © The Royal Society of Chemistry 2025

Supplementary Information

Fluorescent sensor-modified polyvinyl alcohol films for detection of amine vapor based on PET (photo-induced electron transfer)

Kazuki Tao, Keiichi Imato, and Yousuke Ooyama*

Applied Chemistry Program, Graduate School of Advanced Science and Engineering, Hiroshima University, 1-4-1 Kagamiyama, Higashi-Hiroshima 739-8527, Japan
E-mail: yooyama@hiroshima-u.ac.jp

General

^{1}H NMR spectra were recorded using a Varian-500 FT NMR spectrometer. Photoabsorption spectra were observed using a SHIMADZU UV-3600 plus spectrometer. Fluorescence spectra were measured using a Hitachi F-4500 spectrometer. Fluorescence lifetime were measured using a HORIBA Delta Flex spectrometer. Cyclic voltammetry (CV) curves were recorded in acetonitrile/Bu_4NClO_4 (0.1 M) solution with a three-electrode system consisting of Ag/Ag^+ as the reference electrode, a Pt plate as the working electrode, and a Pt wire as the counter electrode using an Electrochemical Measurement System HZ-7000 (HOKUTO DENKO).

Preparation of TF-2-modified PVA film

PVA (605 mg) was added to a mixed solvent of water (24 mL) / EtOH (16.5 mL) and stirred at 70 °C. Once the PVA was completely dissolved and the solution was allowed to return to room temperature. Then, an EtOH solution of DBA (4.1×10^{-2} M) was added to prepare the coating solution. A quartz glass plate treated with piranha acid was immersed in this coating solution and dried to fabricate the PVA/DBA film. This plate was immersed in an EtOH solution of **TF-2** (2.0×10^{-5} M) for 10 minutes, followed by drying and washing with EtOH, resulting in the preparation of the **TF-2-**modified PVA film.

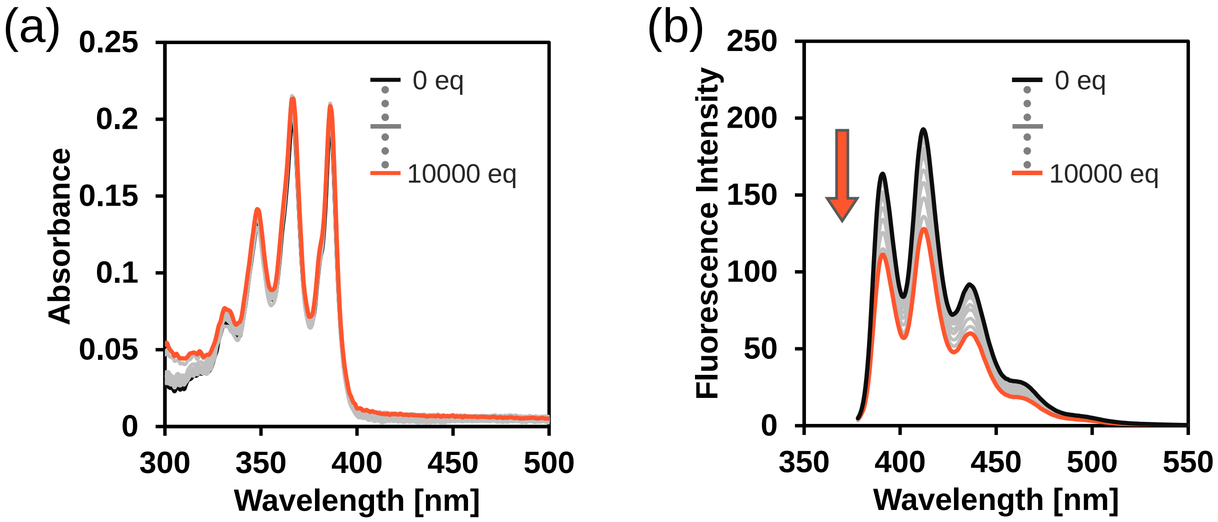

Fig. S1 (a) Photoabsorption and (b) fluorescence spectra (λ^{ex} = 367 nm) of **TF-2** (2.0×10^{-5} M) in acetonitrile containing PDIA.

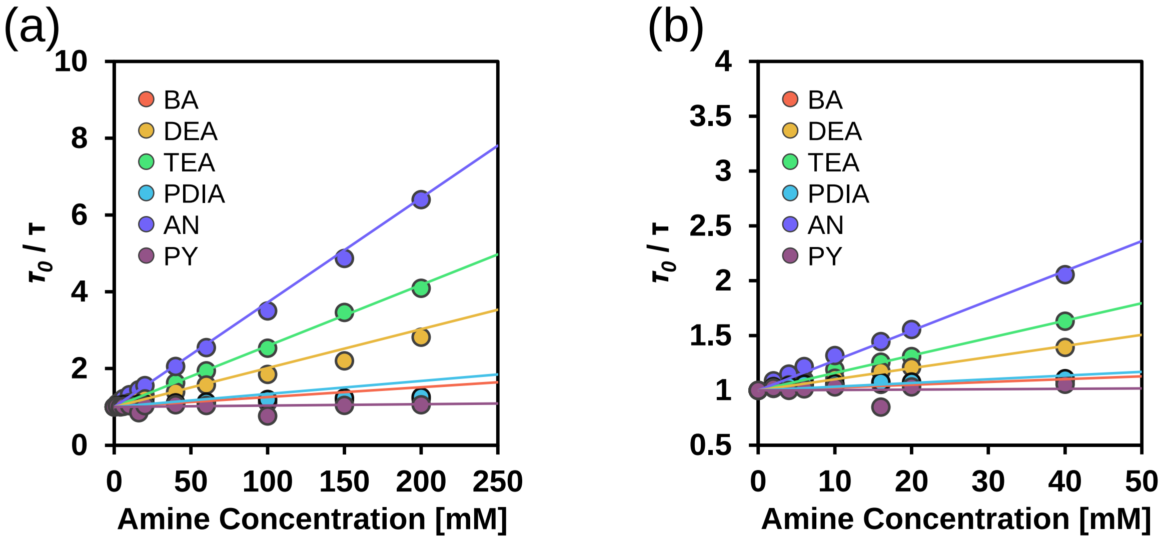

Fig. S2 Relative fluorescence lifetime (τ_0/τ) of **TF-2** (2.0×10^{-5} M) as a function of amine content below (a) 2.5×10^{-1} M and (b) 5.0×10^{-2} M in acetonitrile (λ^{ex} = 367 nm). τ_0 is fluorescence lifetime of **TF-2** solution in the absence of amine.

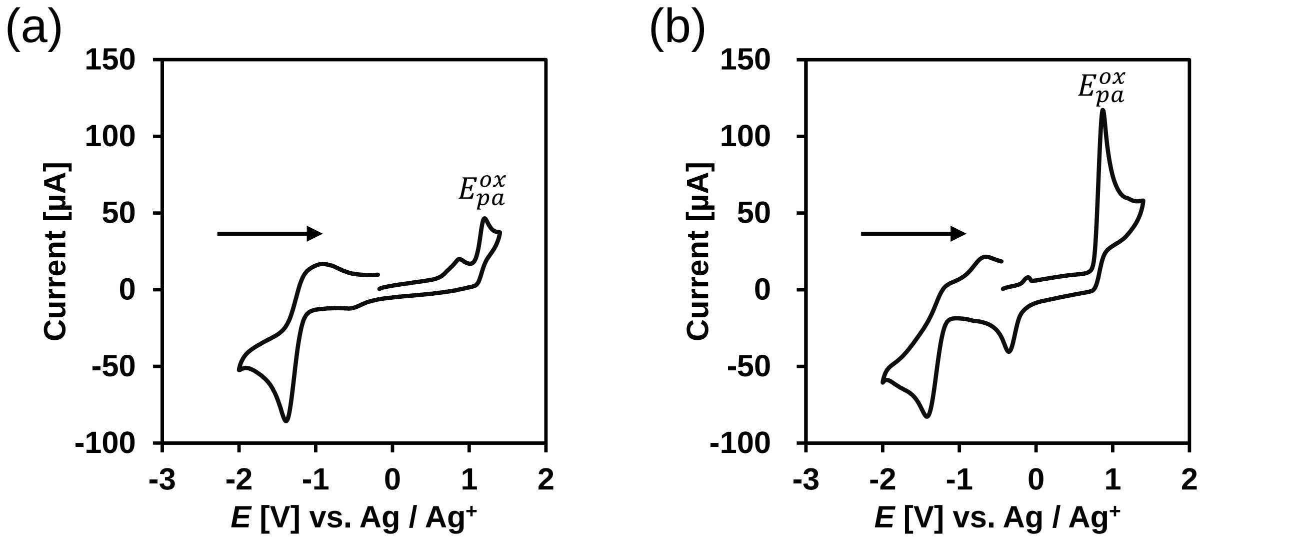

Fig. S3 Cyclic voltammograms of (a) **TF-2** (1.0 mM) and (b) 9-methylanthracene (1.0 mM) in acetonitrile containing 0.1 M tetrabutylammonium perchlorate (Bu_4NClO_4) at scan rate of 100 mV s^{-1}. The E_{pa}^{ox} is attributed to oxidation of anthracene skeleton. The arrow denotes the direction of the potential scan.

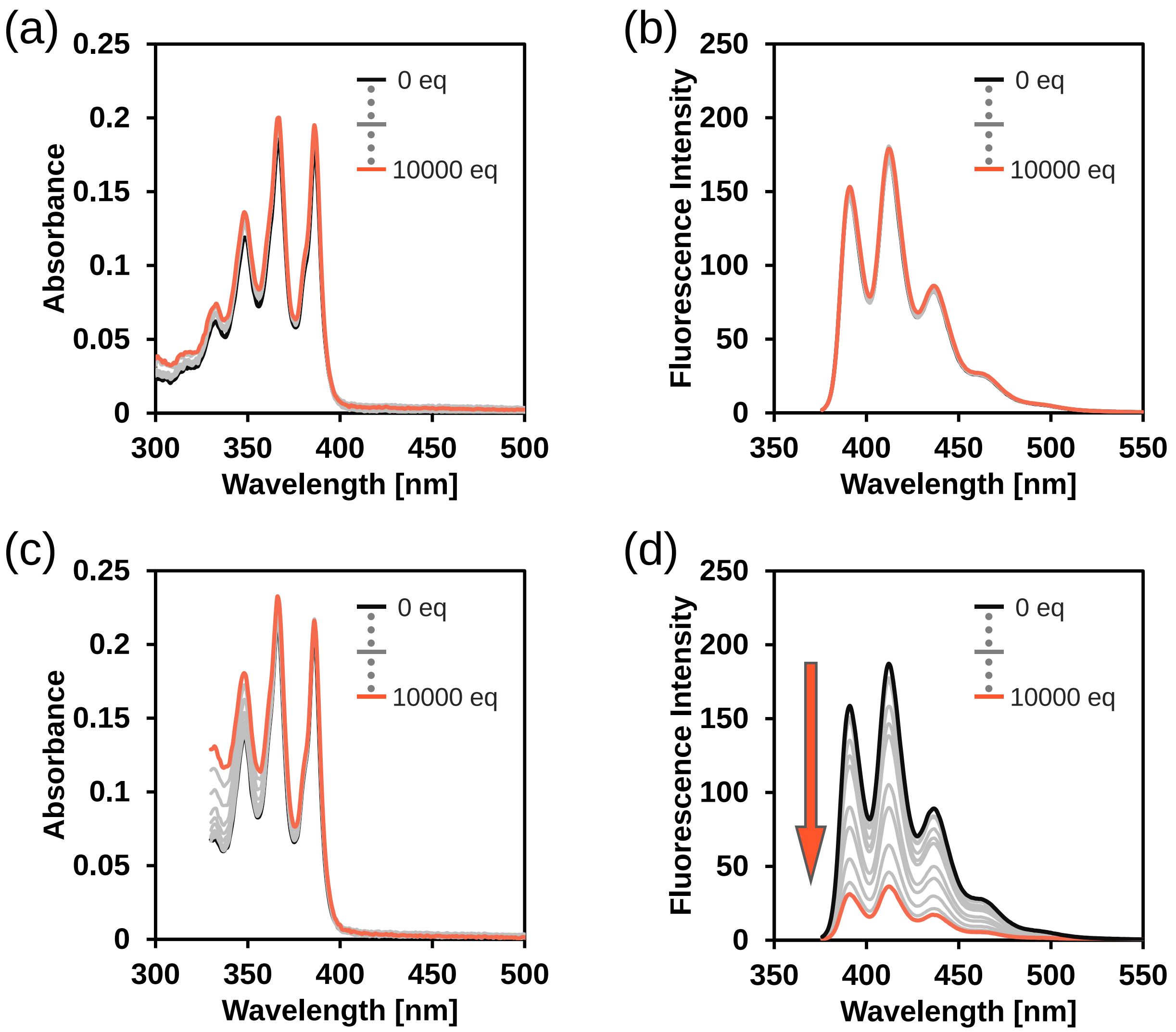

Fig. S4 (a) Photoabsorption and (b) fluorescence spectra (λ^{ex} = 367 nm) of **TF-2** (2.0 × 10^{-5} M) in acetonitrile containing anisole. (c) Photoabsorption and (d) fluorescence spectra (λ^{ex} = 367 nm) of **TF-2** (2.0 × 10^{-5} M) in acetonitrile containing 1,2,4-trimethoxybenzene.

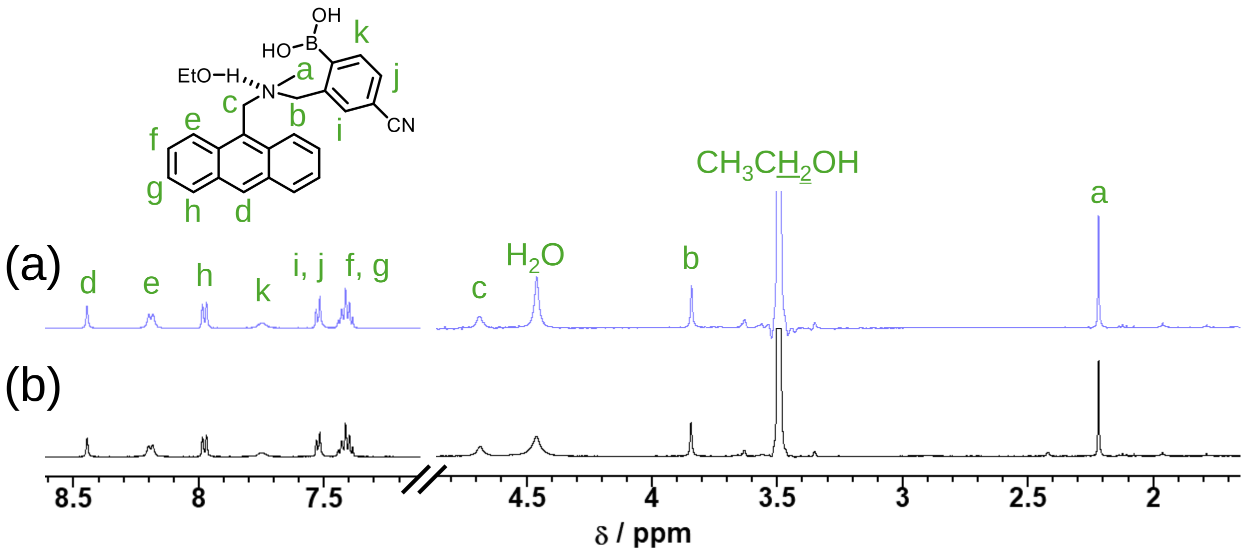

Fig. S5 ^{1}H NMR spectra of **TF-2** (2.0×10^{-4} M) in ethanol-d_6 (a) with and (b) without the addition of TEA-d_{15} (2.0 M).

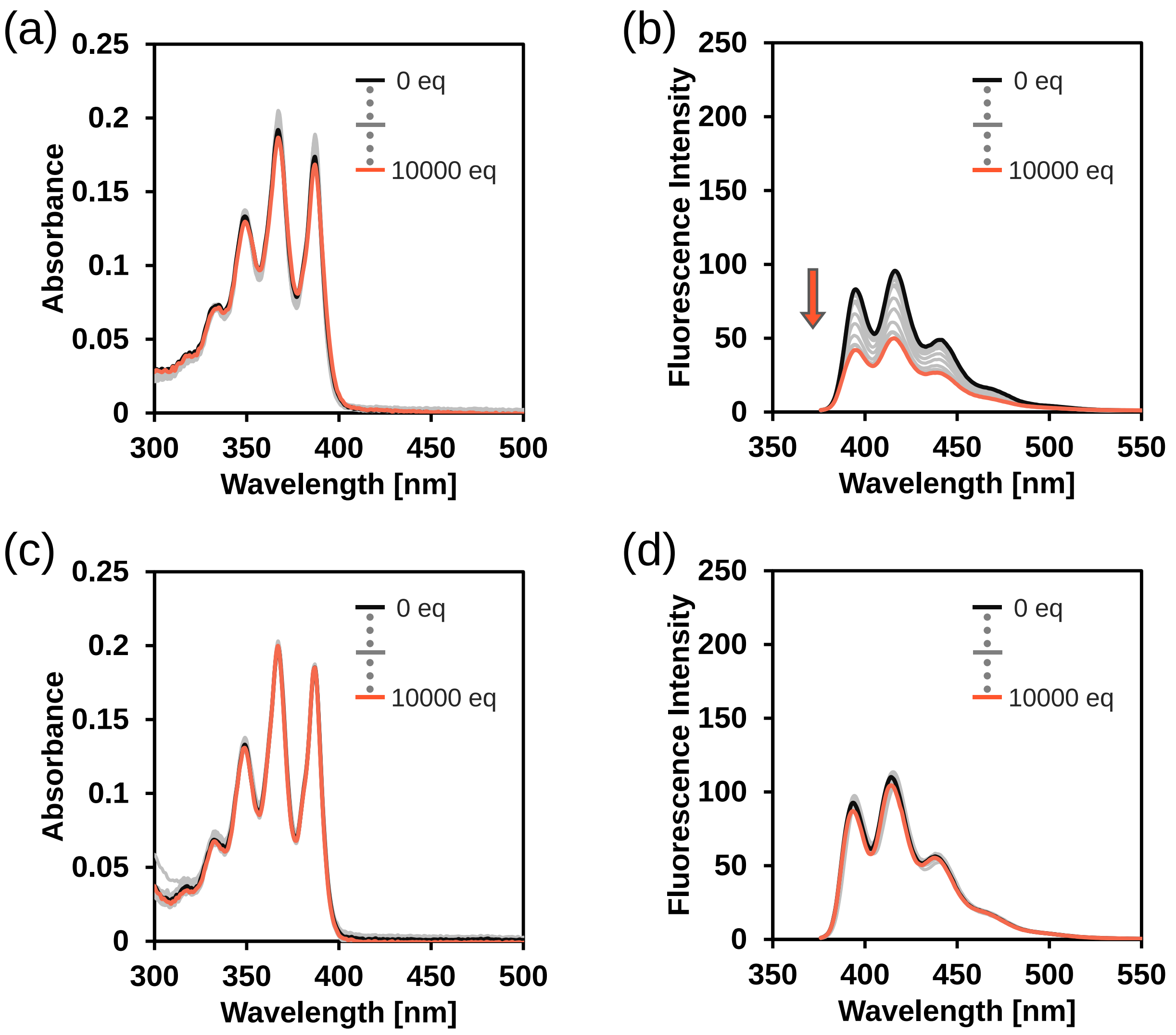

Fig. S6 (a) Photoabsorption and (b) fluorescence spectra (λ^{ex} = 367 nm) of **TF-2** (2.0×10^{-5} M) in ethanol containing TEA. (c) Photoabsorption and (d) fluorescence spectra (λ^{ex} = 367 nm) of **TF-2** (2.0×10^{-5} M) in ethanol containing PY.

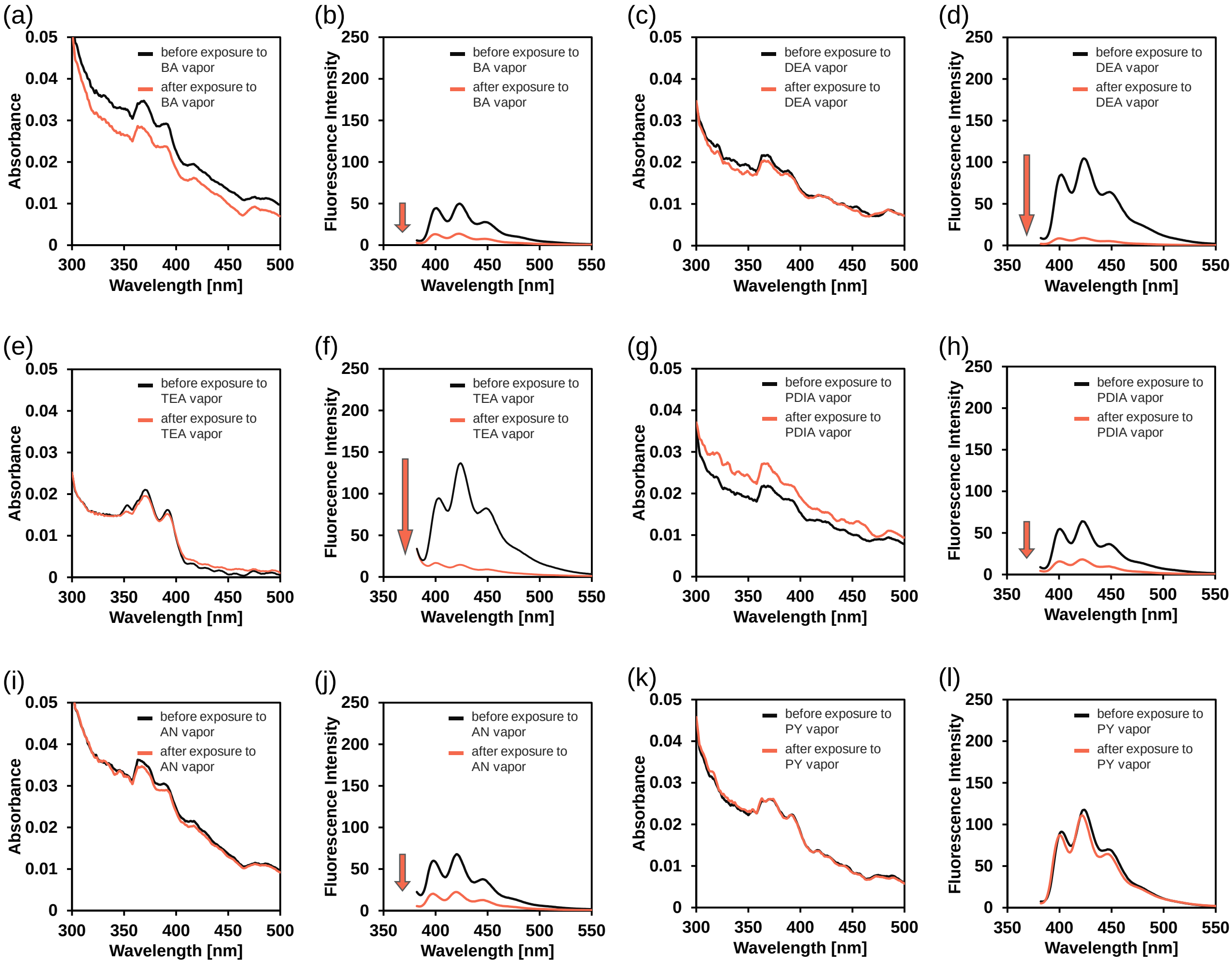

Fig. S7 (a) Photoabsorption and (b) fluorescence spectra (λ^{ex} = 367 nm) of **TF-2**-modified PVA film before and after exposure to BA vapor. (c) Photoabsorption and (d) fluorescence spectra (λ^{ex} = 367 nm) of **TF-2**-modified PVA film before and after exposure to DEA vapor. (e) Photoabsorption and (f) fluorescence spectra (λ^{ex} = 367 nm) of **TF-2**-modified PVA film before and after exposure to TEA vapor. (g) Photoabsorption and (h) fluorescence spectra (λ^{ex} = 367 nm) of **TF-2**-modified PVA film before and after exposure to PDIA vapor. (i) Photoabsorption and (j) fluorescence spectra (λ^{ex} = 367 nm) of **TF-2**-modified PVA film before and after exposure to AN vapor. (k) Photoabsorption and (l) fluorescence spectra (λ^{ex} = 367 nm) of **TF-2**-modified PVA film before and after exposure to PY vapor.

Table S1 Fluorescence lifetime of **TF-2** (2.0×10^{-5} M) in ethanol with and without of TEA or PY.

Sensor	Amine	τ / ns
	-	2.11[a]
**TF-2**	TEA	0.97[b]
	PY	2.72[b]

[a] τ value of **TF-2** solution in the absence of amine. [b] τ value of **TF-2** solution with amine concentration of 2.0×10^{-1} M.
